# Data of characterization of electrospun waste polyethylene terephthalate (PET) nanofibers

**DOI:** 10.1016/j.dib.2020.105535

**Published:** 2020-04-11

**Authors:** Suhad A. Yasin, Jamal A. Abbas, Manaf A. Mohamed, Ibtisam A. Saeed

**Affiliations:** College of Science, University of Duhok, Kurdistan Region, Iraq

**Keywords:** Electrospinning, Nanofiber, Polyethylene terephthalate, Atomic force microscope, Contact angle, X-ray Diffraction, Differential Scanning Calorimetry

## Abstract

The identification and characterization of structural membrane properties, including pores, topography, morphology, and surface interactions, that give essential information for various applications. In this article, we provide a characterization of the electrospinning waste Polyethylene Terephthalate (PET) by using the Atomic Force Microscope (AFM), Contact Angle (CA), X-ray Diffraction (XRD) and Differential Scanning Calorimetry (DSC) of Nanofibers. The data collected in this article is directly related to our previously published research article. The results were obtained to give information associated with the functionalized and surface modification of PET nanofibers.

Specifications table

**Table:** 

Subject	Materials Science
Specific subject area	Characterization of Nanofiber
Type of data	Figures
How data were acquired	Atomic Force Microscope(AFM), Contact Angle,(CA) X-ray Diffraction(XRD), Differential Scanning Calorimetry (DSC).
Data format	Raw
Parameters for data collection	PET concentration (5%), Flow rate (1 ml/h), Voltage (15 kV) and distance needle tip to collector (15 cm).
Description of data collection	Characterization study of the optimized PET Nanofiber after Taguchi experimental design.
Data source location	Institution: University of Duhok City/Town/Region: Duhok/ Kurdistan Region Country: Iraq
Data accessibility	Mendeley Data, V2, doi:10.17632/hj8dtjmz3h.2
Related research article	Yasin, Suhad A., et al. "Methylene blue photocatalytic degradation by TiO2 nanoparticles supported on PET nanofibres." Materials Today: Proceedings (2019) [1].

## Value of the data

• The topography of the surface for PET nanofibers studied by using Atomic Force Microscope (AFM) with the ability to depict and analysis of these surfaces and give the statistical values with high accuracy about surface roughness values depending on the Root Mean Square (RMS) of the average roughness.
• The obtained X-ray Diffraction (XRD) pattern of electrospun PET mats exhibited only an amorphous pattern.
• The contact angle (CA) of electrospun PET nanofiber values is higher than the PET sheet.
• The differential Scanning Calorimetry (DSC) shows a decrease in the melting temperature after electrospinning, which was attributed to the decrease in the overall crystallinity of the electrospun fibers compared to the original polymer.
• Characterization of data that obtained assist the information of physical or chemical modification of the surface, which is related to the applications of PET nanofiber later [2,3].
• The quality of a membrane, for example, hydrophilicity, relies on its surface properties. A hydrophilic membrane is less responsive to fouling and has high water flux during separation [4].

## 1 Data description

The optimization condition to prepare PET nanofiber was PET concentration 5%, Flow rate 1 ml/h, Voltage 15 kV, and distance needle tip to collector 15 cm.

**Fig. 1:**
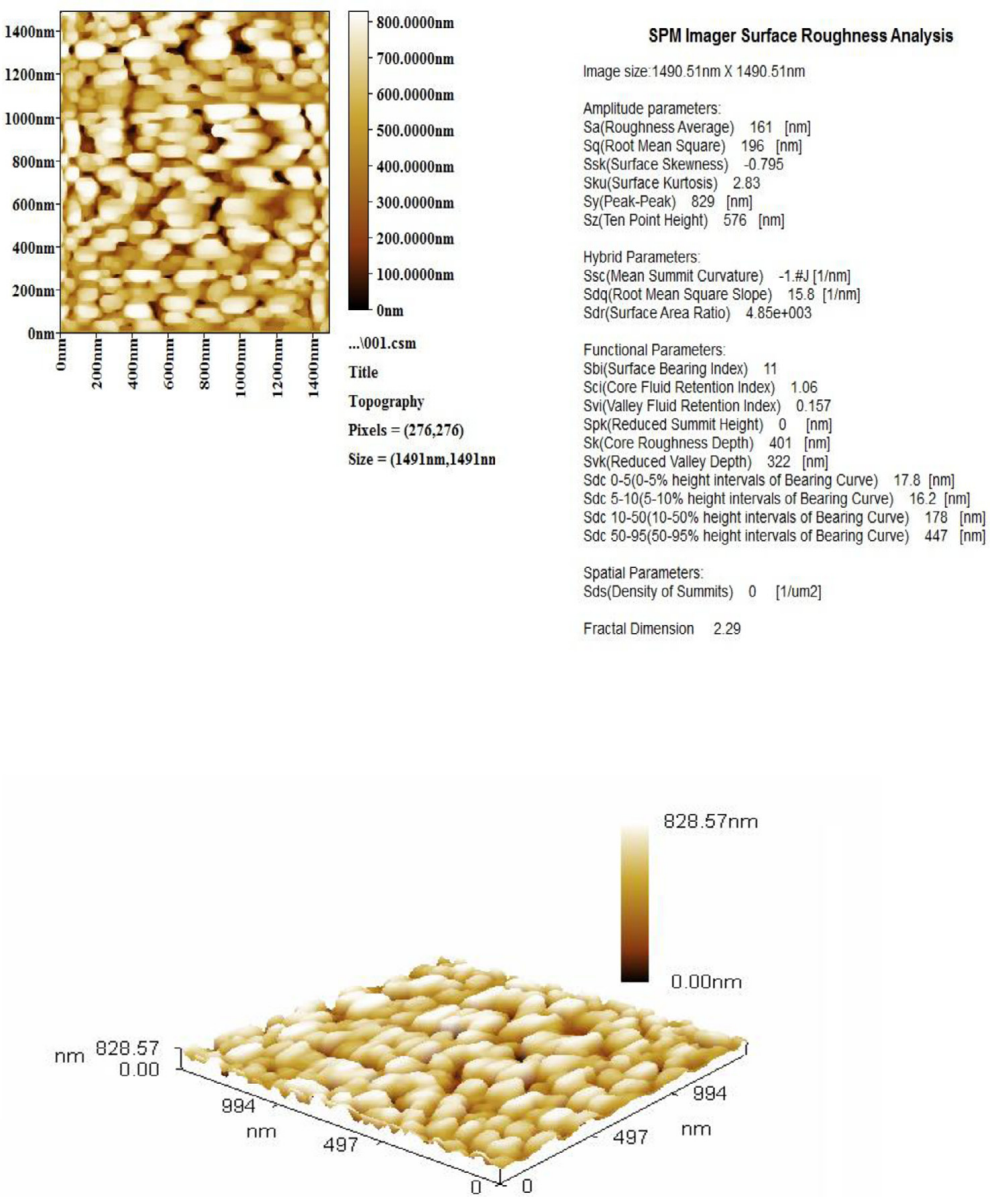
Tridimensional AFM Images. Surface roughness analysis of PET nanofiber, Sa(roughness average) and Sq(root mean square (nm).

**Fig. 2:**
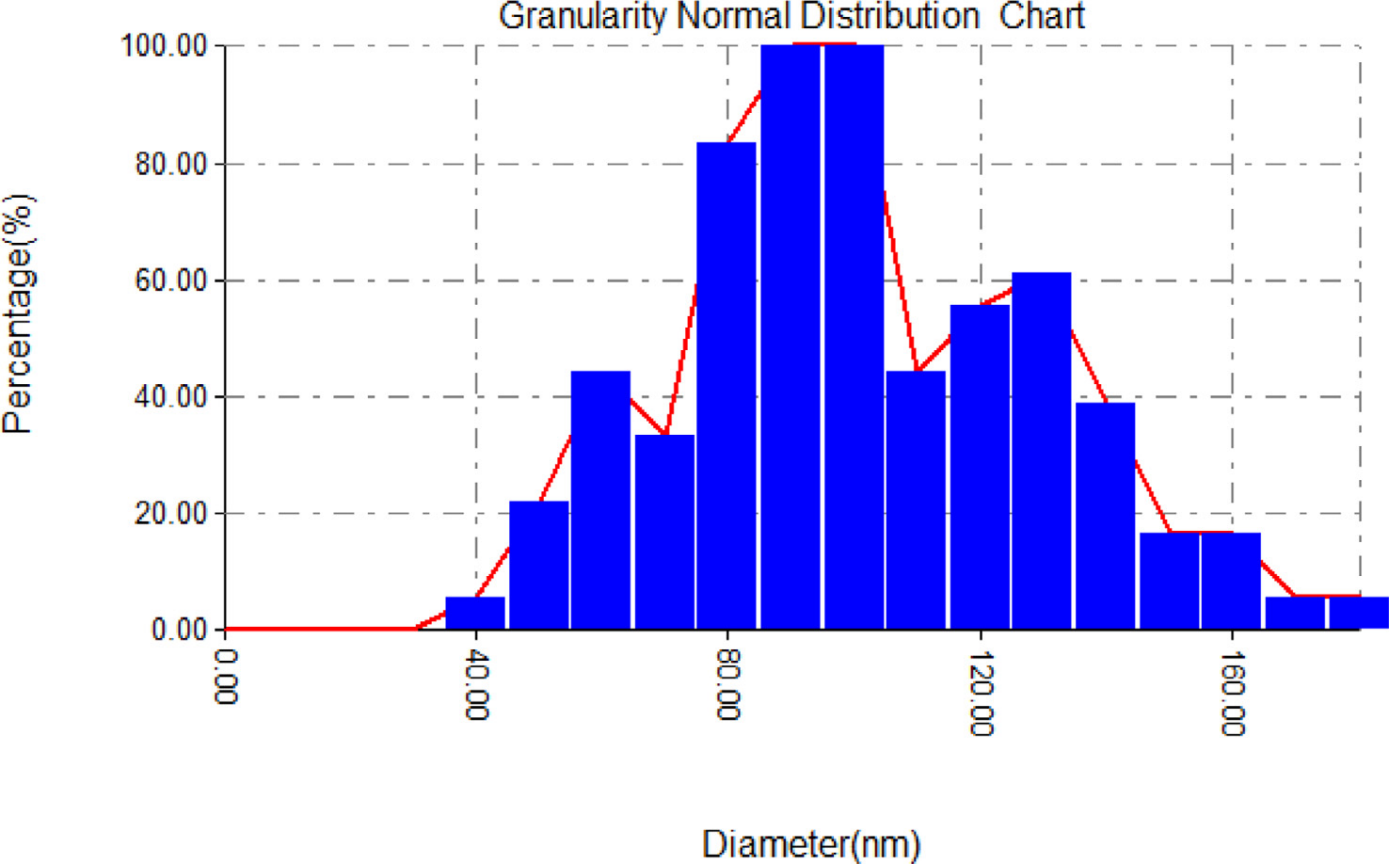
Diameters Size Distribution of Alignment PET Nanofiber. The size distribution shows a wide range of distribution for PET produced by electrospinning by using 5% PET concentration, Flow rate (1 ml/h), Voltage(15 V) and distance needle tip to the collector (15 cm).

The morphology, topography, and membrane roughness characterization of the surface for PET nanofibers were studied by using the Atomic Force Microscope (AFM), as shown in Fig. 1. Also, the diameters size distribution of PET Nanofiber, as displayed in Table 1 and Fig. 2, respectively.

**Table 1:** Granularity normal distribution report.

Avg. Diameter:96.11 nm					<=10% Diameter:50.00 nm			
<=50% Diameter:90.00 nm					<=90% Diameter:130.00 nm			
Diameter (nm)<	Volume (%)	Normal (%)	Diameter (nm)<	Volume (%)	Normal (%)	Diameter (nm)<	Volume (%)	Normal (%)
40.00	0.88	5.56	90.00	15.79	100.00	140.00	6.14	38.89
50.00	3.51	22.22	100.00	15.79	100.00	150.00	2.63	16.67
60.00	7.02	44.44	110.00	7.02	44.44	160.00	2.63	16.67
70.00	5.26	33.33	120.00	8.77	55.56	170.00	0.88	5.56
80.00	13.16	83.33	130.00	9.65	61.11	180.00	0.88	5.56

**Fig. 3:**
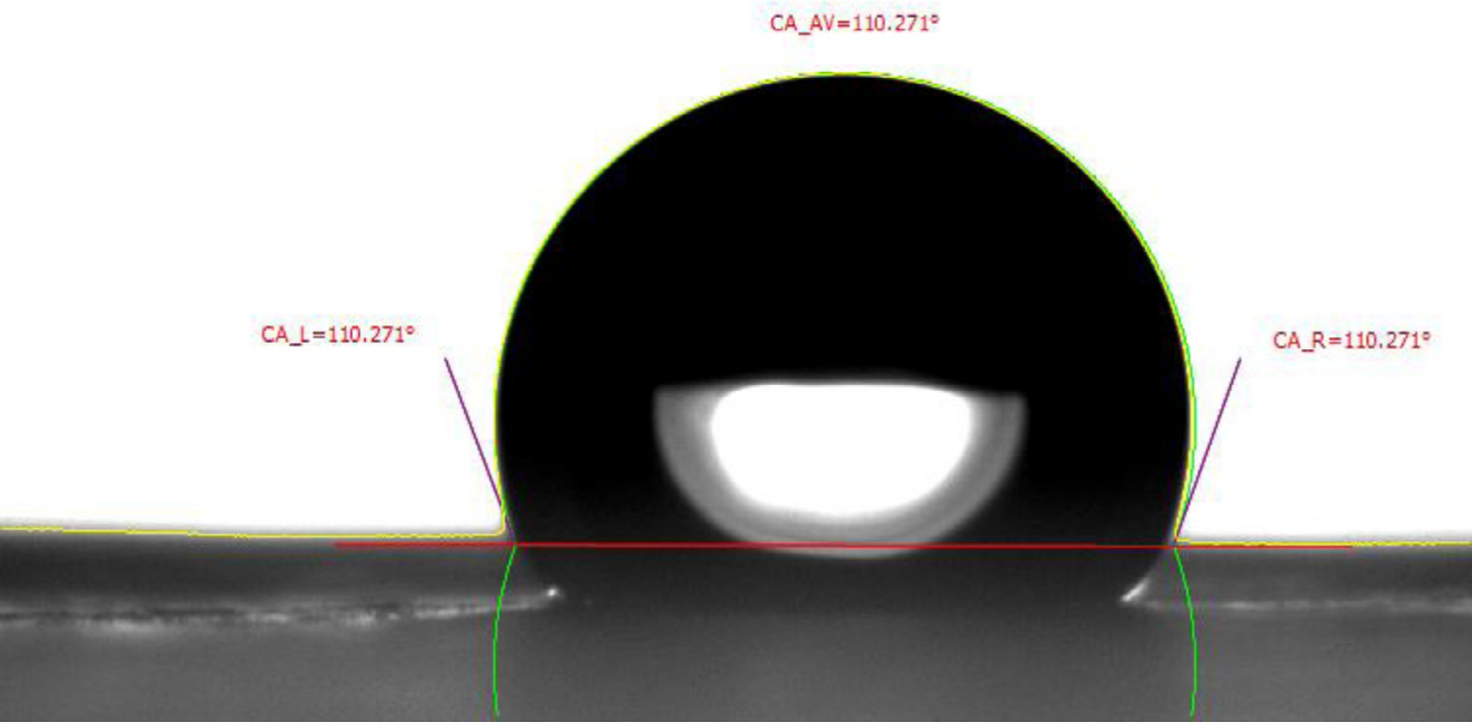
Contact Angle of PET Nanofiber.The surface contact angle influencing by surface roughness which determines by the conditions used.

Surface features like wettability are well known to have a strong dependence on membrane morphology and composition. The contact angle technique is rather basic and straightforward to use the result of PET nanofiber, as shown in Fig. 3.

**Fig. 4:**
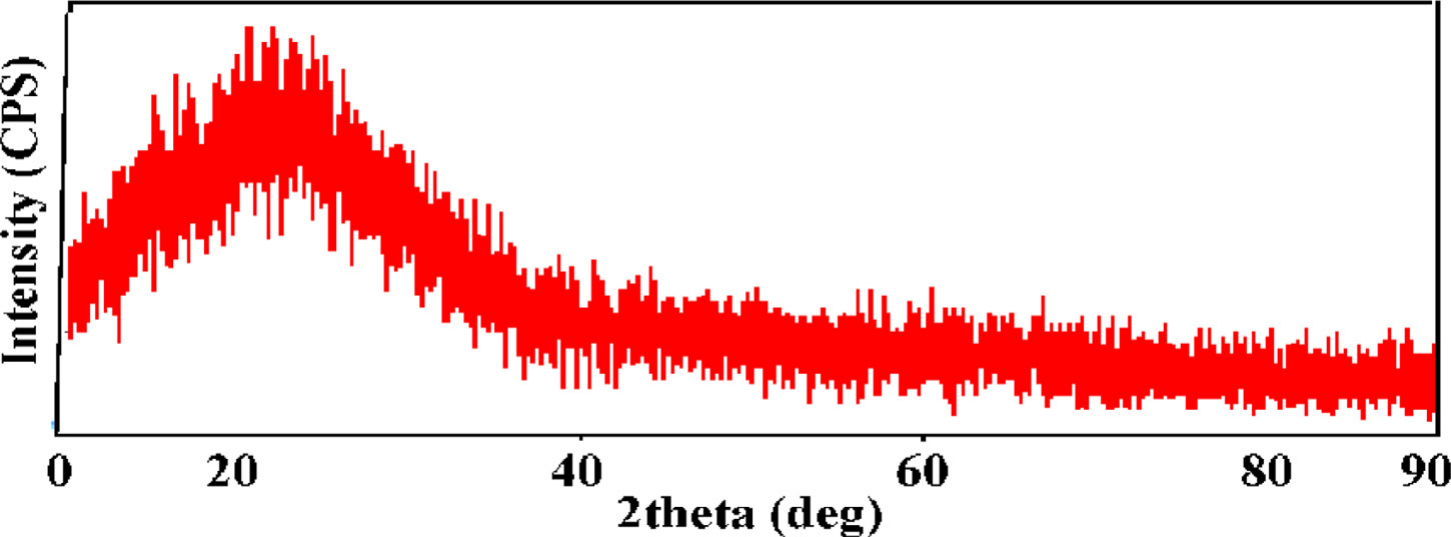
X-ray Diffraction of PET Nanofiber.Abroad peak at (21.05°) related to the amorphous polyester.

The X-ray diffraction was used to analyze the crystallinity of the PET nanofiber; the result of X-ray measurement was represented in Fig. 4.

**Fig. 5:**
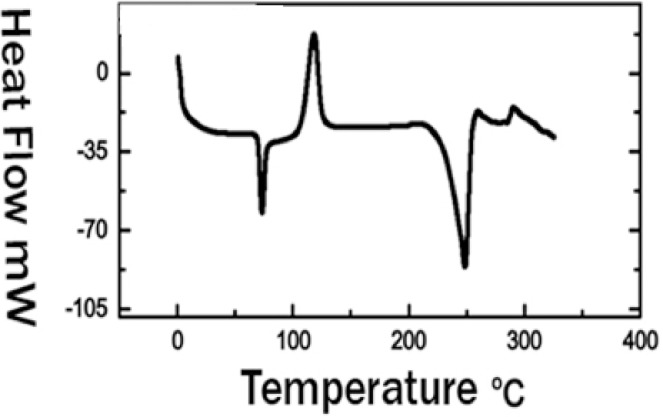
DSC Spectrum of PET Nanofiber .Glass transition, exothermic crystallization, and endothermic melting peaks are typical characteristic peaks.

In DSC curves as shown in Fig 5, the glass transition, cold crystallization, and melting peaks were found in as-spun fibers. PET is a crystallizable polymer because of its regularity in chemical and geometric structures. The levels of crystallinity and morphology significantly affect the properties of the polymers.

## 2 Experimental design, materials, and methods

Through microscopic analysis (AFM), it can be seen from the images that there are fibers that are free from defects such as beads, relatively smooth with a generally uniform thickness along with the fiber, which this related to the electrospinning at low concentration [5]. The parameters of S_a_(roughness average (nm)) is (161), whereas S_q_(root mean square (nm)) is (196), respectively. The diameters size distribution of PET Nanofiber was (140–150 nm).

The results of the static contact angle for the PET nanofiber were collected immediately after depositing the drop of water on its surface. The average result was (110.271°) that is mean, the PET nanofiber has a very rough surface. This result was related to the sample macroscopic because of the very raw hydrophobic materials surface and confronted with the PET nanofibrous samples with the PET sheet [6].

The results of X-ray measurements of the PET nanofiber sample showed a very broad peak at (21.05°) with a d-spacing of (4.128°). It was difficult to calculate the size of the particle-based on the equation of Debeye Sherrer because the material is closer to amorphous. A pick at the (15–20) was related to the polyester molecule [7]. This result is a close agreement with the work reported by Mehdi et al. [8]. The reason is attributed to the stretched PET chains solidified rapidly after elongation, preventing crystal formation in the electrospun PET nanofibers, known for their slow crystallization [9,10].

The electrospun PET matt glass transition near to the anticipated temperature 79 °C was a typical glass transition of the PET. For nanofibre, the peaks of cold crystallization are noted, indicating that the samples contain free amorphous regions. An endothermic peak associated with the fusion of the crystalline fraction appeared about 250 °C. Showing a decrease in the melting temperature after electrospinning, which was attributed to the decrease in the overall crystallinity of the electrospun fibers compared to the original polymer.

## Appendix Supplementary materials

Image, application 1
